# Quantification of Alternative Splicing Variants of Human Telomerase Reverse Transcriptase and Correlations with Telomerase Activity in Lung Cancer

**DOI:** 10.1371/journal.pone.0038868

**Published:** 2012-06-18

**Authors:** Yan Liu, Bing-quan Wu, Hao-hao Zhong, Xin-xia Tian, Wei-gang Fang

**Affiliations:** Department of Pathology, Key Laboratory of Carcinogenesis and Translational Research (Ministry of Education), School of Basic Medical Sciences, Peking University Health Science Center, Beijing, China; University of Texas Southwestern Medical Center, United States of America

## Abstract

Telomerase plays important roles in the development and progression of malignant tumors, and its activity is primarily determined by transcriptional regulation of human telomerase reverse transcriptase (hTERT). Several mRNA alternative splicing variants (ASVs) for hTERT have been identified, but it remains unclear whether telomerase activity is directly associated with hTERT splicing transcripts. In this study, we developed novel real-time PCR protocols using molecular beacons and applied to lung carcinoma cell lines and cancerous tissues for quantification of telomerase activity and three essential hTERT deletion transcripts respectively. The results showed that lung carcinoma cell lines consistently demonstrated telomerase activity (14.22–31.43 TPG units per 100 cells) and various hTERT alternative splicing transcripts. For 165 lung cancer cases, telomerase activity showed significant correlation with tumor differentiation (poorly->moderately->well-differentiated, P<0.01) and with histotypes (combined small cell and squamous cell carcinoma>squamous cell carcinoma>adenosquamous carcinoma>adenocarcinoma, P<0.05). Although the overall hTERT transcripts were detected in all the samples, they were not associated with telomerase activity (r = 0.092, P = 0.24). Telomerase activity was significantly correlated with the transcriptional constituent ratio of α-deletion (r = -0.267, P = 0.026), β-deletion (r = -0.693, P = 0.0001) and γ-deletion (r = –0.614, P = 0.001). The positive rate and average constituent ratio of β-deletion transcripts (92.12%, 0.23) were higher than those of α-deletion (41.82%, 0.12) or γ-deletion (16.36%, 0.18) transcripts. The combined small-cell and squamous cell carcinomas expressed less deletion transcripts, especially β-deletion, than other histotypes, which might explain their higher telomerase activity. In conclusion, the molecular beacon-based real-time PCR protocols are rapid, sensitive and specific methods to quantify telomerase activity and hTERT ASVs. Telomerase activity may serve as a reliable and effective molecular marker to assist the evaluation of histological subtype and differentiation of lung carcinomas. Further studies on hTERT deletion splicing transcripts, rather than the overall hTERT transcripts, may improve our understanding of telomerase regulation.

## Introduction

Normal somatic cells undergo the process of cellular senescence because of progressive telomere shortening after each cell division. Activation of telomerase is believed to be responsible for maintaining sufficient telomeric length in tumor or stem cells. These cells have overcome this proliferative block by expressing the enzyme telomerase, which provides cells with the ability to divide continuously. Thus, regulation of telomerase activity has important implications for many developmental processes including cell proliferation, differentiation and aging as well as tumorigenesis. Telomerase activity has been detected in almost 90% of all human malignancies, making it an obvious target for cancer diagnostic and therapeutic strategies.

The human telomerase reverse transcriptase (hTERT) is an essential component of the holoenzyme complex that adds telomeric repeats to the ends of chromosomes. The expression of normal full-length hTERT correlates well with telomerase activity and seems to be the rate-limiting factor for telomerase activity in human cells. The hTERT gene consists of 16 exons and spans ∼37 kb of genomic DNA, of which ∼33 kb is intronic sequences and the remaining ∼4 kb corresponds to the hTERT mRNA transcript. Recently, three main alternative splicing variants (ASVs) in the reverse transcriptase motifs of hTERT were found to be involved in the control of telomerase activity: i) α-deletion: lacking 36 bp from exon 6 including motif A; ii) β-deletion: lacking 182 bp from exons 7 and 8, leading to a nonsense mutation and truncating the protein before the conserved RT motifs; iii) γ-deletion: lacking 189 bp from exon 11 within motifs D and E [1]. There are several possible combinations of these alternative splicing sites, which result in a large number of potential splicing transcripts, but those with more than two splicing sites are unstable and degraded too fast to be detected. Because α, β and γ deletion transcripts result in truncations or mutations in the reverse transcriptase domain essential for catalytic activity, they are either nonfunctional (β-deletion or γ-deletion) or even have dominant-negative effects (α-deletion) [1]–[4]. Therefore, the correlation between hTERT gene expression and telomerase activity is complicated.

Telomerase activity and hTERT expression were found in 67–85% and 48–95% of lung tumors respectively [5], [6], and both were significantly associated with poor overall survival and disease-free survival in patients with non-small cell lung cancer (NSCLC) [6], [7]. However, the relationships between telomerase activity or hTERT and clinicopathological variables were controversial. Lantuejoul S and coworkers reported that hTERT expression was significantly lower in adenocarcinoma (Adc) than in squamous cell carcinoma (Scc), basaloid carcinoma and small cell lung cancer, and telomerase activity was lower in stage I lung carcinomas than in other stages (II–IV) [5]. Wu TC and coworkers demonstrated that neither telomerase activity nor hTERT expression in NSCLCs was correlated with clinicopathological characteristics such as grade, tumor stages, tumor types or TNM values [8]. Notably, the majority of previous studies tested only telomerase activity or overall hTERT transcripts, and did not distinguish the different splicing variant transcripts. In the present study, a real-time telomere repeat amplification protocol (TRAP) with a reverse primer-linked probe (RPP), a kind of molecular beacon probe combing the reverse primer and fluorescence-labeled probe in one molecule, was developed for telomerase activity quantification in lung tumor cell lines and tissues. Another real-time PCR assay with molecular beacons was developed to analyze the expression levels of the hTERT ASVs in the same samples. Thus we investigated the characterization of telomerase activity and hTERT deletion splicing transcription in lung carcinoma, which may provide important information for the understanding of telomerase regulation in tumorigenesis.

## Methods

### Tumor Material and Cell Lines

Tumor samples were obtained from 165 lung cancer patients (115 men, 50 women; median age, 60 years; range, 31–79 years). Within 10 minutes after surgery, tissues without necrosis and hemorrhage were dissected from the tumor, followed by flash-frozen in liquid nitrogen and stored at −70°C. One part of each sample was subjected to conventional histological examination to ensure that specimens contained at least 80% tumor cells. Diagnosis, grading and pathologic TNM (Tumor, Node, Metastasis) staging were determined independently by two experienced pathologists according to WHO and UICC classification.

The human non-small lung cancer cell lines A549, H1299, SPC-A1 and PAa were used as positive controls for telomerase activity and hTERT gene expression. PAa was established from a Chinese patient with lung adenocarcinoma and kept in our laboratory, while other cell lines were obtained from the American Type Culture Collection (ATCC, Manassas, VA, USA). Cells were grown in Dulbecco’s modified Eagle’s medium (DMEM), supplemented with 100 mL/ L heat-inactivated fetal bovine serum (Gibco BRL, Grand Island, NY, USA), at 37°C under 5% CO_2_. Cells at 80–90% of confluency were harvested by 0.25% trypsin–EDTA and washed with ice-cold phosphate-buffered saline (PBS, pH 7.4). After cell counting, approximately 10^6^ cells were washed twice with PBS and pelleted by centrifugation at 2000 g for 5 min at 4°C. The cell pellets were stored at −70°C for the telomerase assay within six months.

### Ethics Statement

This study was approved by the Peking University Institutional Review Board with the approval No. IRB00001052-10004. All the participants in this study have provided written consent, which has been the review procedure of the ethics committee.

### Primers and Probe Design

**Figure 1:**
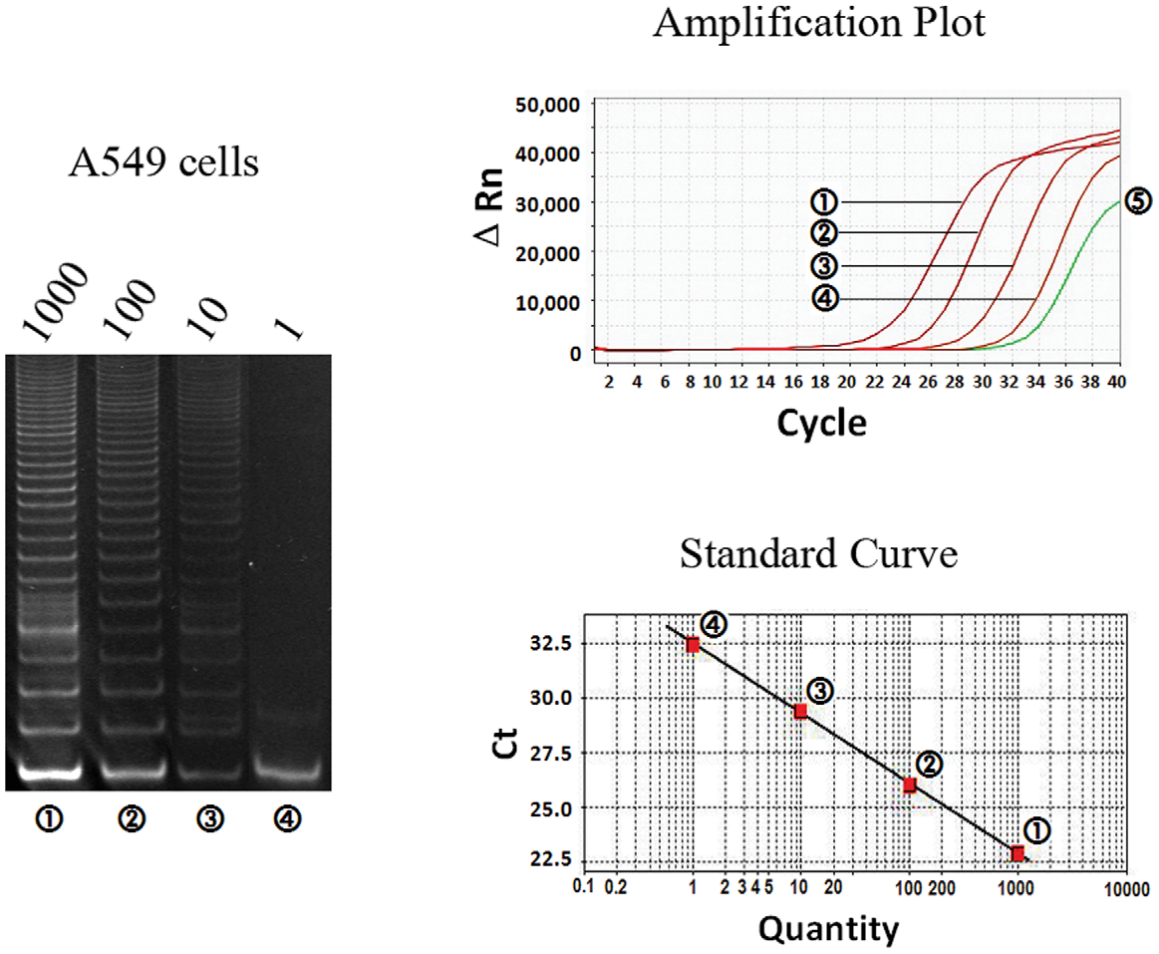
Linearity of the telomeric repeat amplification protocol (TRAP) using reverse primer-linked probe in cancer cell line. Cell extracts equivalent to the indicated numbers of A549 cells were tested for telomerase activity. Results in gel electrophoresis (left), amplification plot (above) and standard curves (below) consistently indicated the linear relationship between the telomerase activity and A549 cell number, while 5 represented a negative control.

The traditional TRAP is a two-step assay in which telomerase adds telomeric repeats onto the 3′-end of the substrate oligonucleotide, and then the extended products are amplified by polymerase chain reaction (PCR) using TS and a reverse primer complementary to the telomeric repeats. In this study, a real-time TRAP assay was developed by using a reverse primer-linked probe (RPP), which combined the reverse primer and fluorescence-labeled probe in one molecule. The principle of this RPP-based real-time TRAP assay is diagrammed in Fig. 1. The reverse primer-linked probe was synthesized by Biosearch Technologies (Novato, CA, USA). Quantification of telomerase activity was accomplished by monitoring the fluorescent signals emitted from the RPPs which had been incorporated into the TRAP products (Figure S1). Molecular beacons for quantification of hTERT overall or deletion transcripts spanned the non-splicing exons or the deletion sites respectively, according to GenBank accession NM_198253 (Figure S2). Primers and probes were designed using the Primer PREMIER 5.0 and OLIGO 6.0 software respectively. Their sequences are presented in Table S1. The delta entropy of the second structure in each probe was calculated by Mfold software (http://www.idtdna.com/Scitools/Applications/mFold/) to check the stability of stem-loop. Beacon probes for hTERT and Taqman probe for the control gene GAPDH (glyceraldehydes-3-phosphate dehydrogenase gene) were synthesized by Sigma-Aldrich (Saint Louis, MO, USA). All primer sets were synthesized and purified by AuGCT Biotechnology (Beijing, China). The specificity of PCR amplifications was assessed by 10% non-denaturing polyacrylamide gel electrophoresis.

### Real-time Quantification Assay of Telomerase Activity

The CHAPS lysis buffer was prepared as described previously [9]. The cell pellets were resuspended in ice-cold lysis buffer (5000 cells/ µL) and incubated for 30 min on ice. After centrifugation (12 000 g, 30 min, 4°C), the supernatants were collected carefully and rapidly frozen at −70°C. The stored tissue samples were partially thawed and approximately 30 mg tissue was minced under sterile conditions until a smooth consistency was obtained. The sample was then transferred to a sterile 1.5 mL micro-centrifuge tube, and mixed with 200 µL CHAPS lysis buffer. RNase inhibitor (Takara Biotechnology, Dalian, China, 100 units/mL) was added to the CHAPS lysis buffer prior to the extraction. The protein concentration was measured by the Bradford method [10]. The final extracts were diluted to a concentration of 2 µg/ µL protein with lysis buffer and immediately stored in aliquots at −70°C.

The total volume of reaction mixture was 20 µL and contained 1×GoTaq flexi buffer, 1.8 mM MgCl_2_, 50 µM each of dNTP, 160 nM modified substrate oligonucleotide primer (MTS), 140 nM reverse primer-linked probe, 1unit of GoTaq DNA polymerase (Promega Corporation, Madison, WI, USA), and 1 µL telomerase extracts. PCR was performed using an ABI StepOne Real-Time Cycler (Applied Biosystems, Framingham, MA, USA). After 30 min incubation at 30°C for telomerase elongation, the reaction mixture was heated at 95°C for 3 min to inactivate the telomerase, followed by a 40-cycle amplification (94°C for 15 sec and 60°C for 60 sec including 10- sec plate reading). Ten microliters of each sample extract was incubated at 95°C for 10 min and then its one-tenth volume was added into another parallel reaction as the heat-inactivation control. In every experiment, 1 µL of CHAPS lysis buffer substituted for protein extract was used as a negative control.

TSR8 is an oligonucleotide identical to the MTS primer extended with eight telomeric repeats AG(GGTTAG)_7_, which can be used as a standard for estimating the amount of MTS primers with telomeric repeats extended by telomerase in a given extract. Furthermore, detection of TSR8 indicates that the amplification step of the TRAP assay was performed effectively [11]. To perform the TRAP assay, 1 µL of each TSR8 dilution (0.2 amol/ µL, 0.04 amol/ µL, 0.008 amol/ µL and 0.0016 amol/ µL, respectively) was used to obtain the standard curve in which 0.001 amol TSR8 corresponds to each unit of TPG (Total Production Generated). For a valid analysis, appropriate controls have been included in every assay:

i) heat-inactivation control: the extract aliquot heated at 95°C for 10 min; ii) negative control: lysis buffer substituted for protein extract and iii) positive control: telomerase-positive cell extracts. Sometimes, positive telomerase activity could only be detected in the diluted extract and cannot be detected in more concentrated extracts because the tissue extract may contain Taq polymerase inhibitors. So for each tissue sample, at least three protein concentrations (2 µg/ µL, 0.2 µg/ µL, 0.02 µg/ µL) were tested in duplicates, in which the highest TPG value was considered as the final telomerase activity.

### RNA Extraction and Real-time RT-PCR Analysis of HTERT ASVs

Total cellular RNA was extracted from frozen samples with the TRIzol reagent (Invitrogen, Carlsbad, CA, USA) according to the manufacturer’s instructions. RNA quality was assessed by denaturing agarose gel electrophoresis. Only samples with sharp 18 S and 28 S rRNA bands and without degradation were used for further analysis. Two microgram RNA from each sample was used for cDNA production using the M-MLV reverse transcriptase and random hexamers (Promega Corporation, Madison, WI, USA). The diluted cDNA (1∶10 dilution) was used for the amplification of control GAPDH to assess reverse transcription efficiency as well as RNA integrity.

Real-time PCR was performed in a total volume of 15 µL containing 1×GoTaq flexi buffer, 5 mM MgCl_2_, 200 µM each of dNTP, 1 unit of GoTaq DNA polymerase, 800 nM primers (forward and reverse), 400 nM probes and 1 µL of diluted cDNA (1∶10 dilution). The annealing temperature was different for different ASV. The cycling protocol consisted of 3 min initial denaturation at 95°C and a 40-cycle amplification (94°C for 15 sec, annealing temperature for 60 sec including plate reading). When testing each ASV transcript with the specific primer set, two transcriptional products, with or without deletion, would be amplified simultaneously, but the Beacon probe only hybridized with the deletion transcriptional product and emitted fluorescence [12]. The Ct value of each hTERT splicing variant was normalized to the Ct-value of GAPDH by subtracting the GAPDH Ct-value from the target Ct value. The relative expression level for each target PCR was calculated using the equation [13]: Relative expression  = 2^-[Ct(target)-Ct(GAPDH)]^×10000. The hTERT ASV constituent ratio (calculated by dividing the relative expression value of the overall transcripts by that of the ASVs) was also determined for each tissue sample.

### Statistical Analysis

Basic descriptive statistical data were obtained using the SPSS 11.0 software. Spearman’s correlation coefficients (r) were calculated to assess associations among telomerase activity, hTERT ASV expression and clinicopathological data. Differences in telomerase activity or quantitative hTERT expression between the analyzed subgroups were evaluated by the unpaired t-test or one-way ANOVA. A p value <0.05 was considered significant.

## Results

### Quantification of Telomerase Activity and HTERT ASVs in Tumor Cell Lines

The sensitivity and linearity of the RPP-based real-time TRAP assay were evaluated with non-small cell lung cancer cell line A549. The cell pellets containing approximately 10^6^ cells were resuspended in 200 µL CHAPS lysis buffer, so the extracts corresponded to telomerase activity of 5000 cells per microliter. The telomerase extracts were serial diluted from 1000 to 1 cell per microliter, and 1 µL extract was used in the quantification of telomerase activity in triplicates. The telomerase in extracts would add telomeric repeats onto the 3'-end of the substrate oligonucleotide (MTS) in the first incubation step and the products were amplified as template in the subsequent PCR process. As shown in Figure 1, using the TRAP-polyacrylamide gel electrophoresis (TRAP-PAGE) assay, telomerase activity could be detected in at least ten A549 cells, indicated by the presence of a visible hexanucleotide ladder of PCR products on the polyacrylamide gel. However, using RPP-based real-time TRAP assay, telomerase activity could be detected in as few as one A549 cell. A linear relationship was also observed between telomerase activity and the logarithm of the number of cells ranging from 1 to 1000 cells. The correlation coefficient calculated from the linear regression model was up to 96.4%. Including telomerase extraction, the RPP-based real-time TRAP assay could be finished within three hours, whereas the TRAP-PAGE would take at least 5 hours. Importantly, the RPP-based real-time TRAP assay was accurately quantitative and reproducible. As shown in Table 1, cell lines A549, H1299, SPC-A1 and PAa consistently demonstrated telomerase activity from 14.22 to 31.43 TPG units per 100 cells.

**Figure 2:**
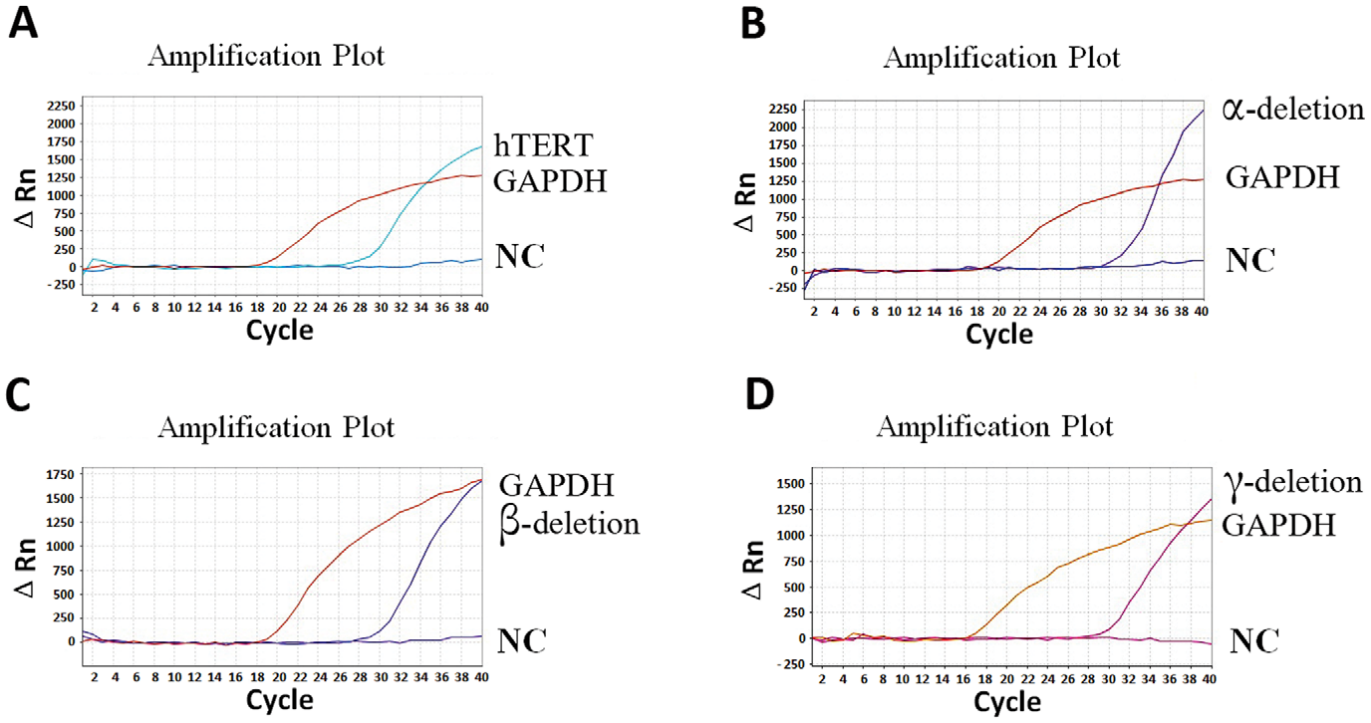
Quantification of hTERT deletion splicing transcripts in SPC-A1 cell line using molecular beacons. Representative linear amplification curves were shown for quantitative detection of overall hTERT (A), α-deletion (B), β-deletion (C) and γ-deletion (D) transcripts respectively. GAPDH served as an internal control. NC: negative control.

Molecular beacons are sensitive and specific because the probes are not hydrolyzed in the amplification and only recognize the perfectly complementary targets. In the quantification of hTERT ASVs, the molecular beacon probe anneals to the corresponding transcripts and monitors the synthesis of the specific nucleic acids in real time. For example, the probe H1810 is complementary to the DNA sequence in the junction of exon 3 and exon 4 of hTERT gene, and would hybridize to all the hTERT transcripts and therefore detect overall hTERT transcripts, including normal and variant splicing transcripts. However, the probe A2173, B2331 and R2699 only detect the α, β or γ deletion transcripts respectively. By using the newly developed quantification protocol, in SPC-A1 cells, all of the three deletion splicing transcripts were detected, with the ratio 0.21, 0.26 and 0.04 for α, β or γ deletion transcripts respectively (Table 1 and Figure 2), which would induce partial nonfunctional transcripts and may explain its low telomerase activity. In other cell lines, the γ-deletion transcript was not detected and the expression levels of α-deletion transcripts (range, 0.25–0.44) were higher than β deletion transcripts (Table 1).

**Table 1:** Quantification of telomerase activity and hTERT alternative splicing variants in tumor cell lines.

Cell lines	Source	Telomerase activity*(TPG units)	hTERT overall transcripts#	hTERT alternative splicing variants						
				Relative expression#			Constituent ratio★			
				α-deletiontranscripts	β-deletiontranscripts	γ-deletiontranscripts	α-deletiontranscripts	β-deletiontranscripts	γ-deletiontranscripts	
A549	Lung adenocarcinoma	31.43±6.47	17.00±2.92	4.25±2.14	2.44±0.68	Not found	0.25	0.14	Not found	
SPC-A1	Lung adenocarcinoma	14.22±9.27	15.14±2.70	3.22±0.81	3.97±0.81	0.61±0.04	0.21	0.26	0.04	
H1299	Lung large cell carcinoma	20.37±2.76	5.61±0.88	2.44±1.62	0.46±0.02	Not found	0.44	0.08	Not found	
PAa	Lung adenocarcinoma	21.42±3.57	14.80±3.73	4.25±1.17	1.98±1.61	Not found	0.29	0.13	Not found	

hTERT: human telomerase reverse transcriptase.

* The means±standard deviation of duplicate determinations.

# The means±standard deviation of triplicate determinations.

★ It was calculated by dividing the relative expression value of the overall transcripts by that of each deletion.

### Quantification of Telomerase Activity and HTERT ASVs in Lung Tumor Samples

**Figure 3:**
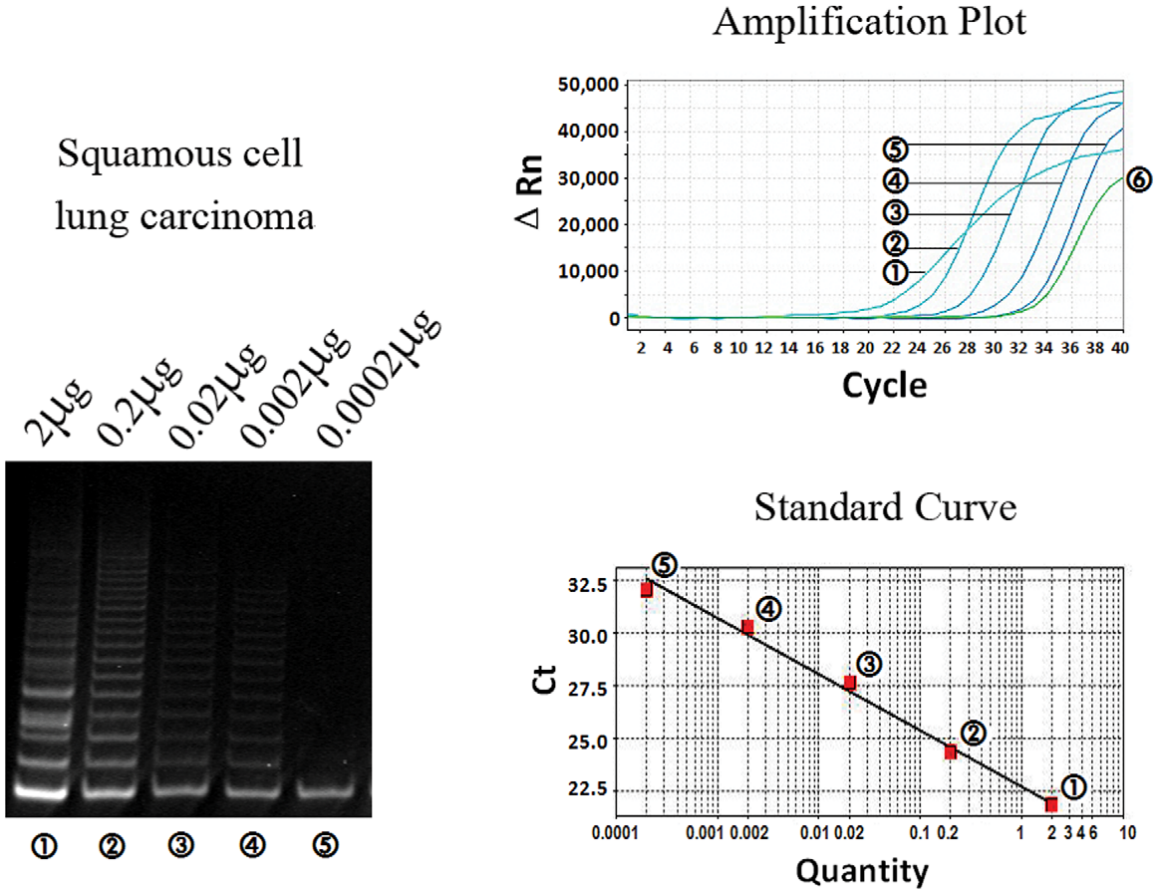
Linearity of the telomeric repeat amplification protocol (TRAP) using reverse primer-linked probe in tumor tissue. Serial diluted protein extracts from 2 µg to 0.0002 µg were tested for telomerase activity in one case of squamous cell carcinoma. Results in gel electrophoresis (left), amplification plot (above) and standard curves (below) consistently indicated the linear relationship between the telomerase activity and protein concentration, while 6 represented a negative control.

In the TRAP assay of tumor tissue sample, the results in both polyacrylamide gel electrophoresis and amplification curves of RPP-based real-time TRAP indicated the linear relationship between the telomerase activity and logarithm of protein concentration (Figure 3). In one case of squamous cell carcinoma, telomerase activity could be detected at a protein concentration as low as nearly 0.002 µg/ µL. To get reliable quantitative TRAP result, at least three protein concentrations (0.02, 0.2 and 2 µg/ µL) in duplicate for each tumor sample were analyzed with two separate experiments.

In all 165 specimens of lung tumor tissues, telomerase activity ranged from 0.39–482.46 TPG units (Table 2). The average telomerase activity in male patients was significantly higher than that in female patients (P = 0.006). Telomerase activity was negatively associated with tumor differentiation (r = 0.022, P = 0.005), in which the poorly differentiated tumors showed the highest telomerase activity. Telomerase activity was significantly different among the four main histological phenotypes of lung carcinomas (P = 0.001), which was also evaluated in pairs. The combined small cell and squamous cell carcinoma (CSS) showed the highest telomerase activity (vs. other three histotypes, P = 0.001), followed by Scc and adenosquamous carcinoma (ASc) (vs. other three histotypes, P = 0.025 respectively), while Adc showed the lowest telomerase activity (vs. other three histotypes, P = 0.001). No association between the telomerase activity and TNM stage was found in this study (P>0.05).

**Table 2:** Quantification of telomerase activity and hTERT alternative splicing variants in lung tumor tissues.

		Telomerase activity*(TPG units)	hTERT overall transcripts#	hTERT alternative splicing variants					
Variable	n			α-deletion transcripts		β-deletion transcripts		γ-deletion transcripts	
				n	Constituentratio★	n	Constituentratio★	n	Constituentratio★
Sex									
Male	115	30.72±19.44	41.92±22.91	50	0.11±0.04	107	0.20±0.11	19	0.09±0.04
Female	50	12.26±9.27	25.24±10.12	19	0.13±0.07	45	0.31±0.14	8	0.39±0.11
Histological subtype									
Adc	43	7.32±4.37	29.24±13.24	12	0.17±0.09	39	0.28±0.16	6	0.41±0.13
Scc	73	29.11±12.95	51.77±17.31	33	0.11±0.05	66	0.19±0.10	14	0.10±0.04
ASc	27	21.82±12.21	19.45±9.39	11	0.09±0.02	26	0.24±0.09	2	0.24±0.10
CSS	12	79.80±33.11	36.14±15.47	10	0.08±0.02	12	0.08±0.02	4	0.06±0.04
Other	10	15.94±7.86	8.74±3.75	3	0.24±0.12	9	0.50±0.30	1	0.33±0.16
Differentiation									
Well	30	8.09±4.77	36.70±14.46	6	0.17±0.06	25	0.22±0.15	4	0.20±0.13
Moderately	105	24.14±10.03	41.34±19.36	43	0.11±0.08	98	0.24±0.11	16	0.20±0.08
Poorly	30	46.35±16.72	20.68±7.98	20	0.12±0.05	30	0.22±0.11	7	0.18±0.09
Stage									
I/II	98	26.56±11.48	20.93±7.33	46	0.13±0.10	92	0.24±0.15	16	0.19±0.09
III/IV	67	23.03±11.09	60.17±27.49	23	0.09±0.06	61	0.22±0.16	11	0.16±0.07
Pleural metastasis									
Yes	83	18.42±9.72	31.37±16.39	31	0.14±0.05	77	0.27±0.18	10	0.20±0.12
No	82	31.91±13.69	42.42±18.35	38	0.10±0.04	75	0.19±0.10	17	0.17±0.05
Lymph node metastasis									
Yes	91	21.56±9.77	46.45±18.47	35	0.10±0.04	82	0.26±0.11	13	0.18±0.11
No	74	29.52±14.72	25.07±10.48	34	0.13±0.09	70	0.20±0.13	14	0.17±0.12

hTERT: human telomerase reverse transcriptase; Adc, adenocarcinoma; Scc, squamous cell carcinoma; ASc, adenosquamous carcinoma; CSS, combined small cell and squamous cell carcinoma.

* The means±standard deviation of duplicate determinations.

# The means±standard deviation of triplicate determinations.

★ It was calculated by dividing the relative expression value of the overall transcripts by that of each deletion.

Quantification of the overall and deletion splicing hTERT transcripts were performed in all of tumor samples. In Spearman’s analysis, there was no significant correlation between the expression of hTERT overall transcripts and telomerase activity (r = 0.092, P = 0.241), and between the expression of hTERT overall transcripts and any clinicopathologic parameter (P>0.05). The α, β and γ deletion transcripts were detected in 41.82%, 92.12% and 16.36% of patients, and their average constituent ratio was 0.12, 0.23 and 0.18 respectively. Only 16 patients expressed all of the three deletion transcripts at the same time. It was remarkable that all of three deletion transcripts’ constituent ratios were significantly correlated with the telomerase activity, while the correlation coefficient was –0.267 for α-deletion transcript (P = 0.026), –0.693 for β-deletion transcript (P = 0.0001) and –0.614 for γ-deletion transcript (P = 0.001), respectively. In male patients, the average constituent ratios of the three deletions were lower than those in female patients, consistent with the higher telomerase activity among them. However, only β-deletion and γ-deletion showed significant difference between male and female patients (P = 0.006, P = 0.027, respectively). As for pairwise comparisons between histologic subtypes and three deletion transcriptional constituent ratios, the CSS showed significant lower constituent ratio in β-deletion (P = 0.011) and γ-deletion (P = 0.015) than Adc. The Scc only presented lower constituent ratio in γ-deletion (P = 0.006) than Adc. There was no significant association between any deletion transcriptional constituent ratio and tumor differentiation, stage or lymph node metastasis. Furthermore, the patients with pleural metastasis showed significant higher transcriptional constituent ratio in β-deletion (P = 0.041), but not in other two deletions (P = 0.227 and P = 0.735 respectively).

## Discussion

Since telomerase is expressed in almost 90% of all human cancers, there has been great interest in developing a telomerase assay suitable for clinical testing [14]. As an end-point and semi-quantitative analysis, the traditional TRAP is low-throughput, limited in accurate quantification and likely to be false negative because the PCR amplification efficiency may be inhibited by the protein in cell extract. Quantification of telomerase activity using real-time analysis is more precise because it relies on Ct values determined during the exponential phase of PCR at relatively low concentrations of PCR product before saturation or plateau. Previous real-time quantitative TRAP with fluorescent dyes or Taqman probe is less specific and more limited in its quantitative potential because of no one-to-one correspondence between fluorescence and PCR product [15], [16]. Here, we described a novel quantitative TRAP assay by using a reverse primer-linked probe (RPP). RPP is a molecular switch to detect DNA amplification by utilizing energy transfer between fluorophore (FAM) and quencher (DABSYL). The OFF to ON transition occurs when the conformation of the RPP changes from a “closed” intramolecular stem-loop structure to an “open” extended structure. This structural change is achieved when one RPP is incorporated into a double-stranded DNA molecule by PCR. The amplification can be monitored by directly measuring the fluorescence of the reaction mixture. In this study, telomerase activity demonstrated satisfactory linear relationships with the logarithm of cultured cell number or protein concentration in proper range. Telomerase activity could be detected in as few as one cultured tumor cell or as low as 0.002 µg protein from tumor tissue. Consistent with previous report, the fluorescein-probe-based real-time TRAP assay gave more rapid, accurate results for telomerase activity quantification than traditional TRAP with gel electrophoresis [17]. In our previous study, we examined the telomerase activity of 60 lung cancer tissues using SYBR Green real-time TRAP, a relative quantitative analysis, and only found the difference in telomerase activity between NSCLC and CSS [9]. In this study, we applied the RPP-based real-time TRAP to a larger number of lung tumor tissue samples (165 cases) and used standard curve analysis. A significant difference in telomerase activity among four histological types was found: CSS> Scc > ASc > Adc, which was consistent with Fujiwara’s report [18]. Ohmura and Maniwa have reported higher telomerase activity in NSCLC (42.3 and 75.24 TPG units respectively), in which they examined 60 and 40 cases respectively, and used semi-quantitative methods [19], [20]. We consider that such contradictions could be attributed to diversity in populations and sample size, and especially to the different techniques used to detect telomerase activity. It was also conflicting in literatures whether the level of telomerase activity was correlated with the aggressive clinicopathological features, such as tumor differentiation and TNM stage [6], [19]–[21]. Using the RPP-based real-time TRAP, we observed a strong correlation between telomerase activity and tumor differentiation. It was interesting that difference in telomerase activity between male and female patients was observed in this study. That female patients mainly suffered from Adc, which had lower telomerase activity than other subtypes, may contribute to their lower telomerase activity than male patients. Thus, telomerase activity appears to be a useful marker in determining histological type and differentiation in lung carcinomas.

Some authors claimed that the detection of hTERT mRNA by RT-PCR or in situ hybridization could be used to evaluate the telomerase activity [5]. However, the transcriptional and posttranscriptional regulation of hTERT was complex and not fully elucidated. Previously, primer set or Taqman probe spanning the deletion site was used in most studies for splicing variant examinations [22]–[26]. However, these primer sets or probes might amplify both the full-length transcripts and deletion transcripts because of the staggered annealing with mixed templates. In the present study, we developed a quantitative assay using molecular beacons and detected all of three deletion transcripts in SPC-A1 cells. We found a relative high constituent ratio (range, 0.21–0.44) for the α-deletion transcripts in the four lung cancer cell lines, which is not consistent with the data showing lower constituent ratio (range, 0.06–0.15) previously [1], [25], [27]. This may be attributed to our use of more specific molecular beacon, rather than the semi-quantitative nested PCR assays. The patterns of hTERT ASVs were shown to be varied in breast, liver, gastric, colon, thyroid and lung tumors, in which the positive rates of hTERT ASVs were probably overestimated by amplifying a region spanning both deletion sites [1], [11], [21]–[24], [26], [28]. Our quantitative assay for each deletion transcript provides a solution to accurately quantify the ASV expression and evaluate its relationship with clinic-pathological parameters.

In this study, no association between the overall hTERT transcripts expression and telomerase activity was observed, consistent with previous reports in other tumors [29], [30], which indicated that it was not reasonable to substitute the overall hTERT mRNA detection for telomerase activity evaluation. We found that more than 92% of patients expressed at least one deletion transcription, but only 9.7% expressed all of the three deletion transcripts. Notably, the constituent ratios of the three deletion transcripts were significantly correlated with telomerase activity. The β-deletion transcripts were the most common splicing variants in most lung carcinomas and showed the strongest correlation with telomerase activity. This is similar to the previous findings in other tumor types [11], [22]–[24], [26]. Our results showed that the constituent ratios of deletion transcripts, especially the β-deletion, were less in CSS than in NSCLC, which might explain CSS’ higher telomerase activity. The three deletion ASVs didn’t show any correlation with tumor differentiation, stage or lymph node metastasis. Interestingly, we found that pleural metastasis group presented significant higher β-deletion transcript, which could be due to the high percentage of Adc patients in this group. The fact that there is a significant heterogeneity in the expression of hTERT ASVs raises many questions about the possible role of the alternate transcripts of hTERT in the regulation of telomerase and in malignant transformation. Alternative splicing is not random and often inhibits the expression of hTERT protein containing functional reverse transcriptase domains. Telomerase regulation by its splicing variants might be cell type-specific or histotype-specific, as suggested by the precise patterns of splicing that occur in particular cell types during development [3]. In two cases of adenocarcinomas, we did not find any deletion transcript, whereas, we found lower telomerase activity (<1.5 TPG unit) and higher expression of overall hTERT transcripts (>15). A possible explanation for this discordance could be the existence of other mechanism of transcriptional regulation, such as insertions. The complication in the regulation of hTERT transcription in lung carcinoma raised some questions in selecting suitable markers to evaluate telomerase activity. The real-time TRAP assay may be a quantitative, reproducible and time-efficient testing tool to directly evaluate telomerase activity of clinical tumor tissues. When the fresh or frozen tissue is not available to test telomerase activity, the detection of the hTERT transcripts including the overall and deletion ASVs, may be a suitable marker for telomerase activity.

In conclusion, we have developed highly specific, sensitive and rapid quantitative PCR protocols for determination of telomerase activity and hTERT deletion transcripts. Quantification of the telomerase activity may serve as a reliable and effective tool to assist the evaluation of histological subtype and differentiation of lung carcinomas. Further research on hTERT ASVs, rather than the overall hTERT transcript, may improve our understanding of telomerase activity regulation.

## Supporting Information

Figure S1 **Principle of the quantitative TRAP with the reverse primer-linked probe (RPP), which combines the reverse primer and fluorescence-labeled probe in one molecule and is a kind of molecular beacon.** At the initial incubation step, telomeric repeats are added to the 3′ end of the substrate primer (MTS) by active telomerase. In the preceding PCR cycles, the RPP anneals to the 3′ end of telomerase extension product in the reverse direction. The annealing temperature and time allows the fast priming reaction to be finished sufficiently and accurately in the exact complementary site. After this step, both ends of the products are not the telomeric repeats, but complementary to the MTS and RPP. RPP is a molecular switch to detect DNA amplification by utilizing energy transfer between fluorophore (FAM) and quencher (Dabsyl). The OFF to ON transition occurs when the conformation of the RPP changes from a “closed” intra-molecular stem-loop structure to an “open” extended structure. This structural change is achieved when one RPP is incorporated into a double-stranded DNA molecule by PCR. The amplification can be monitored by directly measuring the fluorescence of the reaction mixture. In this assay, the fluorescent signal is only observed after PCR amplification of telomerase-specific products, thereby eliminating non-specific background.
(TIF)

Figure S2 **Principle of the detection of hTERT deletion splicing transcripts by molecular beacons.** (Top) Locations of telomerase-specific T motif, seven conserved reverse transcriptase motifs (1, 2, A, B’, C, D and E), exons 3–13 and deletion sites are indicated. (Bottom) In the absence of hTERT deletion splicing transcriptional product, the probe does not emit fluorescence, as the quencher is close to the fluorophore in the stem-loop structure. Hybridization of the probe sequences with the splicing product separates the quencher from the fluorphore and restores fluorescence.
(TIF)

Table S1 **Primers and probes used in real-time quantification of telomerase activity and hTERT transcription.**
(DOC)

## References

[1] Hisatomi H, Ohyashiki K, Ohyashiki JH, Nagao K, Kanamaru K (2003). Expression profile of a γ-Deletion variant of the human telomerase reverse transcriptase gene.. Neoplasia.

[2] Colgin LM, Wilkinson C, Englezou A, Kilian A, Robinson MO (2000). The hTERT α splice variant is a dominant negative inhibitor of telomerase activity.. Neoplasia.

[3] Ulaner GA, Hu JF, Vu TH, Giudice LC, Hoffman AR (2001). Tissue-specific alternate splicing of human telomerase reverse transcriptase (hTERT) influences telomere lengths during human development.. Int J Cancer.

[4] Wick M, Zubov D, Hagen G (1999). Genomic organization and promoter characterization of the gene encoding the human telomerase reverse transcriptase (hTERT).. Gene.

[5] Lantuejoul S, Soria JC, Moro-Sibilot D, Morat L, Veyrenc S (2004). Differential expression of telomerase reverse transcriptase (hTERT) in lung tumors.. Br J Cancer.

[6] Marchetti A, Pellegrini C, Buttitta F, Falleni M, Romagnoli S (2002). Prediction of survival in stage I lung carcinoma patients by telomerase function evaluation.. Lab Invest.

[7] Wang L, Soria JC, Kemp BL, Liu DD, Mao L (2002). hTERT expression is a prognostic factor of survival in patients with stage I non-small cell lung cancer.. Clin Cancer Res.

[8] Wu TC, Lin P, Hsu C, Huang Y, Chen C (2003). Loss of telomerase activity may be a potential favorable prognostic marker in lung carcinomas.. Lung Cancer.

[9] Liu Y, Wu BQ, Zhong HH, Xu ML, Fang WG (2010). Detection of telomerase activity in cultured cells and tumor tissue of lung carcinoma by modified telomeric repeat amplification protocol.. Pathol Int.

[10] Bradford MM (1976). A rapid and sensitive method for the quantitation of microgram quantities of protein utilizing the principle of protein-dye binding.. Anal Biochem.

[11] Piotrowska K, Kleideiter E, Mürdter TE, Taetz S, Baldes C (2005). Optimization of the TRAP assay to evaluate specificity of telomerase inhibitors.. Lab Invest.

[12] Taveau M, Stockholm D, Spencer M, Richard I (2002). Quantification of splice variants using molecular beacon or scorpion primers.. Anal Biochem.

[13] Hartmann U, Brümmendorf T, Balabanov S, Thiede C, Illme T (2005). Telomere length and hTERT expression in patients with acute myeloid leukemia correlates with chromosomal abnormalities.. Haematologica.

[14] Kim NW, Piatyszek MA, Prowse KR, Harley CB, West MD (1994). Specific association of human telomerase activity with immortal cells and cancer.. Science.

[15] Hou M, Xu D, Björkholm M, Gruber A (2001). Real-time quantitative telomeric repeat amplification protocol assay for the detection of telomerase activity.. Clin Chem.

[16] McGruder BM, Atha DH, Wang W, Huppi K, Wei WQ (2006). Real-time telomerase assay of less-invasively collected esophageal cell samples.. Cancer Lett.

[17] Elmore LW, Forsythe HL, Ferreira-Gonzalez A, Garrett CT, Clark GM (2002). Real-time quantitative analysis of telomerase activity in breast tumor specimens using a highly specific and sensitive fluorescent-based assay.. Diagn Mol Pathol.

[18] Fujiwara M, Okayasu I, Takemura T, Tanaka I, Masuda R (2000). Telomerase activity significantly correlates with chromosome alterations, cell differentiation, and proliferation in lung adenocarcinoma.. Mod Pathol.

[19] Ohmura Y, Aoe M, Andou A, Shimizu N (2000). Telomerase activity and Bcl-2 expression in non-small cell lung cancer.. Clin Cancer Res.

[20] Maniwa Y, Yoshimura M, Obayashi C, Inaba M, Kiyooka K (2001). Association of p53 gene mutation and telomerase activity in resectable non-small cell lung cancer.. Chest.

[21] Wang J, Liu X, Jiang W, Liang L (2000). Telomerase activity and expression of the telomerase catalytic subunit gene in non-small cell lung cancer: correlation with decreased apoptosis and clinical prognosis.. Chin Med J (Engl).

[22] Xu JH, Wang YC, Geng X, Li YY, Zhang WM (2009). Changes of the alternative splicing variants of human telomerase reverse transcriptase during gastric carcinogenesis.. Pathobiology.

[23] Wang Y, Kowalski J, Tsai HL, Marik R, Prasad N (2008). Differentiating alternative splice variant patterns of human telomerase reverse transcriptase in thyroid neoplasms.. Thyroid.

[24] Rha SY, Jeung HC, Park KH, Kim JJ, Chung HC (2009). Changes of telomerase activity by alternative splicing of full-length and beta variants of hTERT in breast cancer patients.. Oncol Res.

[25] Lincz LF, Mudge LM, Scorgie FE, Sakoff JA, Hamilton CS (2008). Quantification of hTERT splice variants in melanoma by SYBR green real-time polymerase chain reaction indicates a negative regulatory role for the beta deletion variant.. Neoplasia.

[26] Mavrogiannou E, Strati A, Stathopoulou A, Tsaroucha EG, Kaklamanis L (2007). Real-time RT-PCR quantification of human telomerase reverse transcriptase splice variants in tumor cell lines and non-small cell lung cancer.. Clin Chem.

[27] Yi X, Shay JW, Wright WE (2001). Quantitation of telomerase components and hTERT mRNA splicing patterns in immortal human cells.. Nucleic Acids Res.

[28] Saebøe-Larssen S, Fossberg E, Gaudernack G (2006). Characterization of novel alternative splicing sites in human telomerase reverse transcriptase (hTERT): analysis of expression and mutual correlation in mRNA isoforms from normal and tumour tissues.. BMC Mol Biol.

[29] Büchler P, Conejo-Garcia JR, Lehmann G, Müller M, Emrich T (2001). Real-time quantitative PCR of telomerase mRNA is useful for the differentiation of benign and malignant pancreatic disorders.. Pancreas.

[30] Hara T, Noma T, Yamashiro Y, Naito K, Nakazawa A (2001). Quantitative analysis of telomerase activity and telomerase reverse transcriptase expression in renal cell carcinoma.. Urol Res.

