# Supplementary figures and images for: Quantification of Alternative Splicing Variants of Human Telomerase Reverse Transcriptase and Correlations with Telomerase Activity in Lung Cancer
Source: PLoS One. 2012 Jun 18;7(6):e38868. doi: 10.1371/journal.pone.0038868 (PMC3377688; doi:10.1371/journal.pone.0038868)

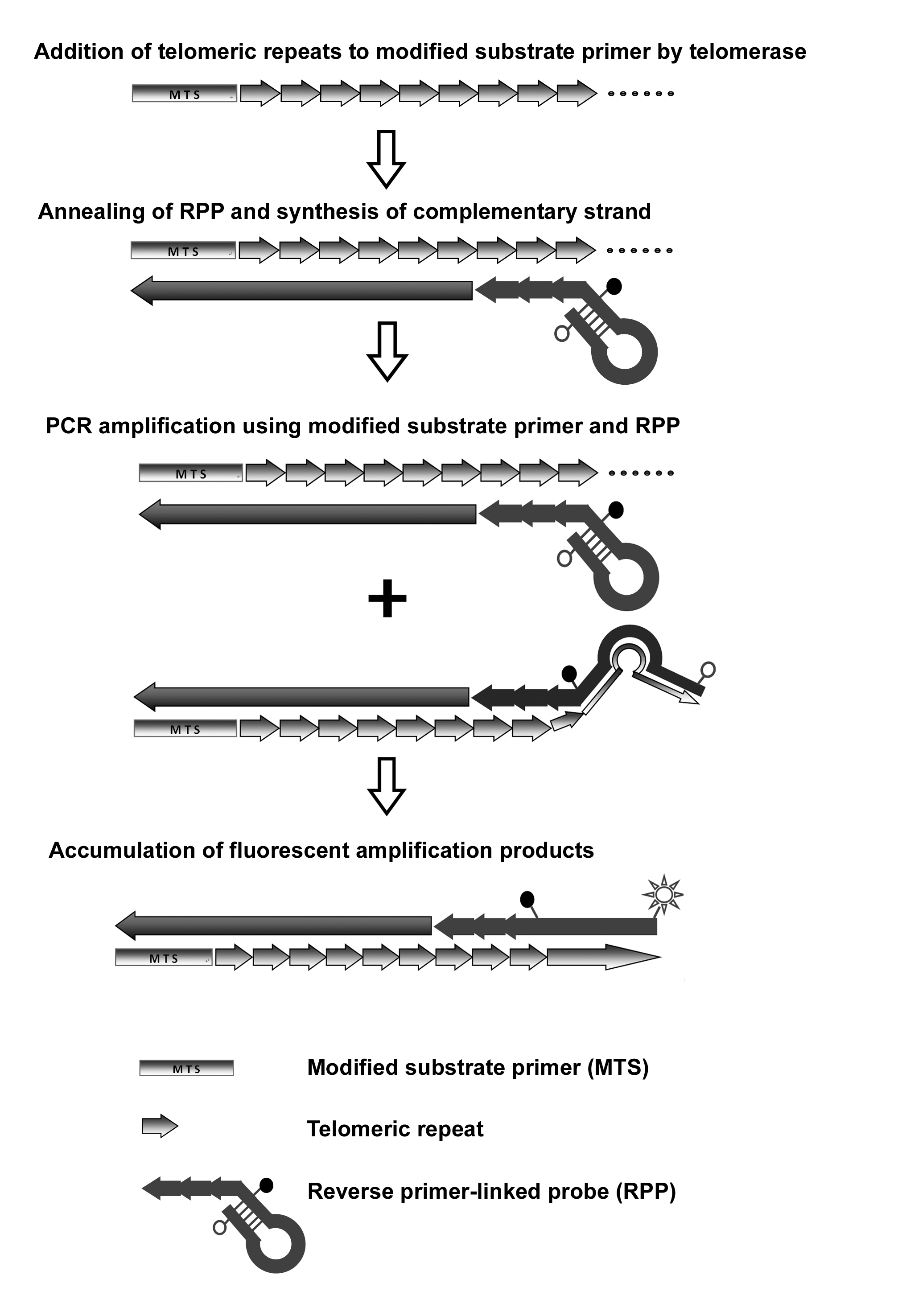

Supplement: Figure S1 — Principle of the quantitative TRAP with the reverse primer-linked probe (RPP), which combines the reverse primer and fluorescence-labeled probe in one molecule and is a kind of molecular beacon. At the initial incubation step, telomeric repeats are added to the 3′ end of the substrate primer (MTS) by active telomerase. In the preceding PCR cycles, the RPP anneals to the 3′ end of telomerase extension product in the reverse direction. The annealing temperature and time allows the fast priming reaction to be finished sufficiently and accurately in the exact complementary site. After this step, both ends of the products are not the telomeric repeats, but complementary to the MTS and RPP. RPP is a molecular switch to detect DNA amplification by utilizing energy transfer between fluorophore (FAM) and quencher (Dabsyl). The OFF to ON transition occurs when the conformation of the RPP changes from a “closed” intra-molecular stem-loop structure to an “open” extended structure. This structural change is achieved when one RPP is incorporated into a double-stranded DNA molecule by PCR. The amplification can be monitored by directly measuring the fluorescence of the reaction mixture. In this assay, the fluorescent signal is only observed after PCR amplification of telomerase-specific products, thereby eliminating non-specific background. (TIF) [file pone.0038868.s001.tif]

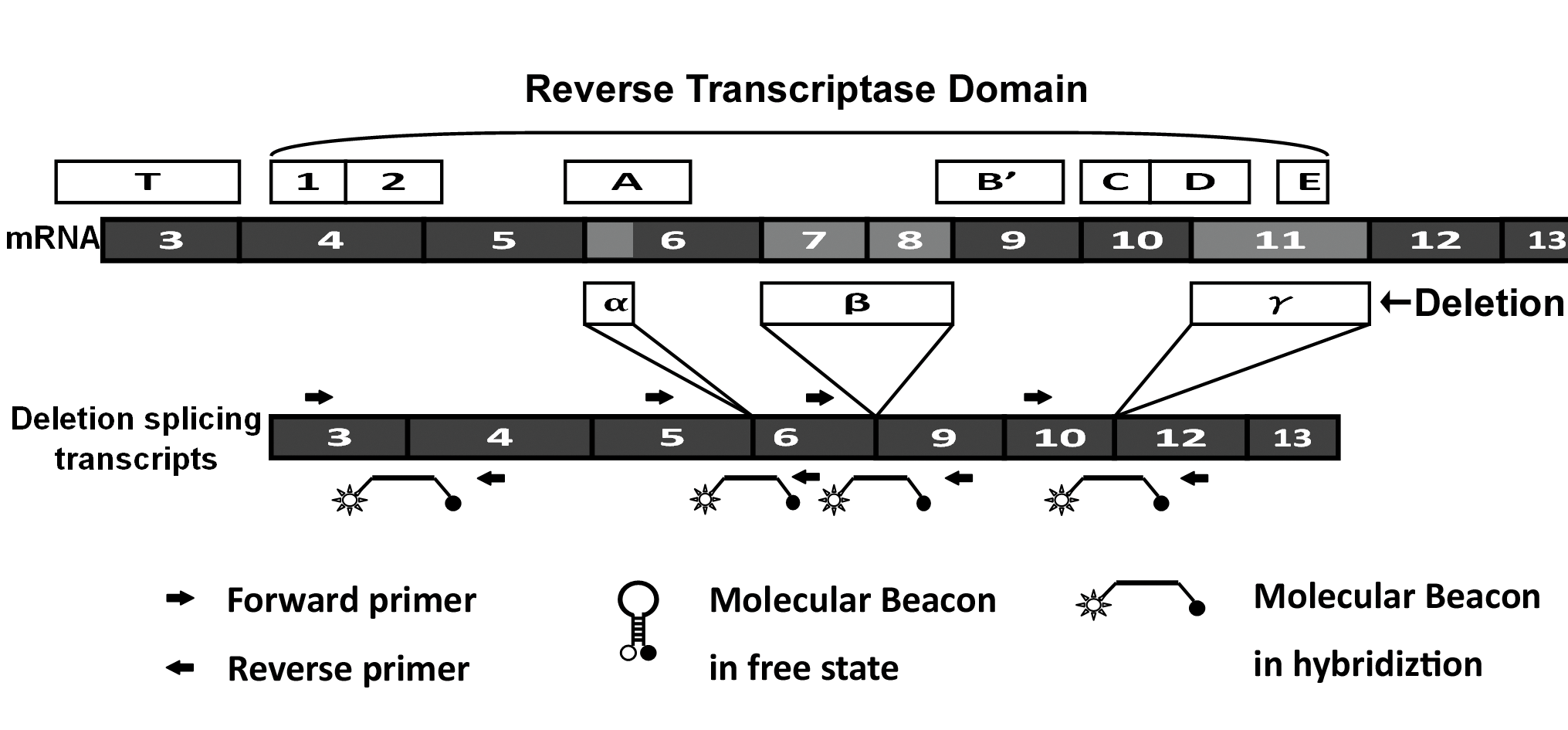

Supplement: Figure S2 — Principle of the detection of hTERT deletion splicing transcripts by molecular beacons. (Top) Locations of telomerase-specific T motif, seven conserved reverse transcriptase motifs (1, 2, A, B’, C, D and E), exons 3–13 and deletion sites are indicated. (Bottom) In the absence of hTERT deletion splicing transcriptional product, the probe does not emit fluorescence, as the quencher is close to the fluorophore in the stem-loop structure. Hybridization of the probe sequences with the splicing product separates the quencher from the fluorphore and restores fluorescence. (TIF) [file pone.0038868.s002.tif]
