# Supplementary material for: Quantification of Alternative Splicing Variants of Human Telomerase Reverse Transcriptase and Correlations with Telomerase Activity in Lung Cancer
Source: PLoS One. 2012 Jun 18;7(6):e38868. doi: 10.1371/journal.pone.0038868 (PMC3377688; doi:10.1371/journal.pone.0038868)
Supplement: Table S1 — Primers and probes used in real-time quantification of telomerase activity and hTERT transcription. (DOC) [file pone.0038868.s003.doc]

**Table S**1 Primers and probes used in real-time quantification of telomerase activity and hTERT transcription

| Name | Sequence (5’→3’) | Annealing temperature(℃ ) | Comment |
| --- | --- | --- | --- |
| Telomerase activity | |  |  |
| MTS | AGCATCCGTCGAGCAGAGTT | 60 | Substrate primer of telomeric addition |
| RPP | FAM-ACGCGTATATTCGCG-T(Dabsyl)-GGTTACCCTTACCCTTACCCTAACC |  | Reverse primer and fluorescent probe |
|  |  |  |  |
| hTERT transcription | |  |  |
| H1773 | TCTTTTTCTACCGGAAGAGTG | 58 | Forward primer in exon 3 |
| H1884 | CGATGCTGCCTGACCTCT |  | Reverse primer in exon 4 |
| H1810 | [6FAM]CCGGGCCAAGTGCTGTCTGATTCCAATGCTTGCCCGG[BHQ1] |  | Probe for detecting overall transcripts |
|  |  |  |  |
| A2100 | GCCTGGACGATATCCACA | 56 | Forward primer in exon 5 |
| A2258 | GTTTGATGATGCTGGCG |  | Reverse primer in exon 6 |
| A2173 | [6FAM]CGGCCGCGTGAGCCTGTCCTTGACAAAGTACCGGCCG[BHQ1] |  | Probe for detecting α deletion transcripts |
|  |  |  |  |
| B2257 | ACCCCAGAACACGTACTG | 58 | Forward primer in exon 6 |
| B2576 | GCAGCGTGGAGAGGAT |  | Reverse primer in exon 9 |
| B2331 | [6FAM]CGCGGTGGACGTAGGACGTGGCTCTTGACCGCG[BHQ1] |  | Probe for detecting β deletion transcripts |
|  |  |  |  |
| R2940 | CGGTTGAAGGTGAGACTG | 54 | Forward primer in exon 10 |
| R2645 | CTGCGTTTGGTGGATGATT |  | Reverse primer in exon 12 |
| R2699 | [6FAM]CCGCGTCCGGGCATAGCTGAGGAAGGTACGCGG[BHQ1] |  | Probe for detecting γ deletion transcripts |
|  |  |  |  |
| GAPDH control | |  |  |
| G46 | CGACAGTCAGCCGCATCT | 54-58 | Forward primer |
| G172 | TAAAAGCAGCCCTGGTGAC |  | Reverse primer |
| GAPDH | [ROX]AAGGTGAAGGTCGGAGTCAACGGATTTG[BHQ2] |  | Probe for detecting GAPDH transcripts |

MTS, modified telomerase substrate; RPP, reverse primer-linked probe; hTERT, human telomerase reverse transcriptase; GAPDH, lyceraldehydes-3-phosphate dehydrogenase.
